# Galectin-3 sensitized melanoma cell lines to vemurafenib (PLX4032) induced cell death through prevention of autophagy

**DOI:** 10.18632/oncotarget.24516

**Published:** 2018-02-16

**Authors:** Silvina Odete Bustos, Gustavo José da Silva Pereira, Renata de Freitas Saito, Cristiane Damas Gil, Daniela Bertolli Zanatta, Soraya Soubhi Smaili, Roger Chammas

**Affiliations:** ^1^ Instituto do Câncer do Estado de São Paulo, Faculdade de Medicina de São Paulo, São Paulo, Brazil; ^2^ Department of Pharmacology, Federal University of São Paulo, São Paulo, Brazil; ^3^ Laboratory of Histology, Department of Morphology and Genetics, Federal University of São Paulo, São Paulo, Brazil

**Keywords:** autophagy, galectin-3, melanoma, vemurafenib, starvation

## Abstract

Melanoma is a current worldwide problem, as its incidence is increasing. In the last years, several studies have shown that melanoma cells display high levels of autophagy, a self-degradative process that can promote survival leading to drug resistance. Consequently, autophagy regulation represents a challenge for cancer therapy. Herein, we showed that galectin-3 (Gal-3), a β-galactoside binding lectin which is often lost along melanoma progression, is a negative regulator of autophagy in melanoma cells. Our data demonstrated that Gal-3^low/negative^ cells were more resistant to the inhibition of the activity of the cancer driver gene *BRAF*^V600E^ by vemurafenib (PLX4032). Interestingly, in these cells, starvation caused further LC3-II accumulation in cells exposed to chloroquine, which inhibits the degradative step in autophagy. In addition, Gal-3 ^low/negative^ tumor cells accumulated more LC3-II than Gal-3 ^high^ tumor cells *in vivo*. Resistance of Gal-3^low/negative^ cells was associated with increased production of superoxide and activation of the Endoplasmic Reticulum (ER) stress response, as evaluated by accumulation of GRP78. Pharmacological inhibition of autophagy with bafilomycin A reversed the relative resistance of Gal-3^low/negative^ cells to vemurafenib treatment. Taken together, these results show that the autophagic flux is dependent on Gal-3 levels, which attenuate the prosurvival role of autophagy.

## INTRODUCTION

Galectin-3 (Gal-3) belongs to a superfamily of carbohydrate-binding proteins (lectins), and it is characterized to bind with higher specificity to β-galactoside containing oligosaccharides. Gal-3 is constituted by three domains: a short NH_2_-terminal domain, a proline-rich collagen-α-like domain, and COOH-terminal carbohydrate-recognition domain (CRD) [1]. Gal-3 is a pleiotropic protein that mediates processes like cell adhesion, proliferation, death, migration, survival, angiogenesis and metastasis in several tumors. Gal-3 plays different functions, depending on its subcellular location (cell surface, cytoplasm, nucleus, endosomal compartment and mitochondria) and type of tumor [2, 3]. Gal-3 displays both CRD-dependent and independent functions, which makes this molecule versatile to act in diverse scenarios [4, 5].

Melanoma is the most aggressive form of skin cancer and its incidence has increased in the last years. Melanoma progression is a multistage process that may include all the following pigmented lesions; benign nevus, dysplastic nevus, radial and vertical growth phase melanoma and the lethal metastatic melanoma. Although progression from benign lesions is considered rare, the progression model has proven useful in the identification of several molecular lesions that are common in the more advanced cases. Metastatic melanoma can progress and acquire resistance to therapy, besides accumulating a high degree of genotypic heterogeneity [6–8]. Based in accumulating insights on melanoma aggressiveness and resistance, several studies led to the characterization of progression biomarkers, which in turn led to the identification of mechanisms associated with melanoma progression. In line with this, the relationship between Gal-3 expression and melanoma has been explored in the last years. The data indicate that thin primary melanomas express more Gal-3 than benign nevus, and this profile is further lost along tumor progression, leading to a decrease of Gal-3 expression in thicker and metastatic melanoma [9, 10].

During melanoma progression several processes and pathways, such as autophagy, are activated in melanoma cells, leading to the organization of an adaptive response against different types of cellular stress [11, 12]. Autophagy is a complex process involved with cancer initiation, progression and therapy responses [13–15]. The catabolic pathway is essential to maintain the homeostasis through the lysosomal degradation of macromolecules, allowing for increased availability of amino acids, fatty acids and nucleotides for anabolic processes [16, 17]. This pathway has a dual role in cancer. Initially, it was considered a mechanism for tumor-suppression, since disruption of genes related to autophagy, such as beclin-1, resulted in tumor development [18, 19]. Moreover, autophagy prevents cellular damage due to reactive oxygen species (ROS) and maintains energy levels. Defective autophagy leads to chronic oxidative stress, tissue damage, inflammation and genomic instability [15, 20]. Taken together, these data showed that autophagy was able to prevent tumor initiation, but along tumor progression, it promotes tumor cell survival and further growth. Recently, autophagy has also been related with the conditioning of the pre-metastatic niche suggesting that autophagy promotes several steps in the metastatic cascade [21]. Consistently, several studies have shown evidence of autophagy changes in melanoma progression. Metastatic melanoma cells have higher beclin-1 and LC3 expression than primary tumors and also present higher levels of autophagic flux [22, 23]. Furthermore, autophagy pathways at the advanced step of melanoma progression are very important to melanoma survival and chemoresistance [24, 25]. Based on this, deeper studies on the mechanisms of autophagy in melanoma are still necessary to reveal autophagy modulators and pathways associated in order to find new therapeutic targets [11, 26].

As Gal-3 expression is often decreased in more advanced melanomas, phases in which autophagy play a crucial role for cell survival, we hypothesized that Gal-3 would exert a negative control of the autophagic process. Herein, we have tested this hypothesis and investigated the impact of Gal-3 expression in human melanoma cell death pathways under treatment with vemurafenib.

## RESULTS

### Galectin-3 silencing increases resistance of melanoma cells to vemurafenib (PLX-4032) treatment

**Figure 1:**
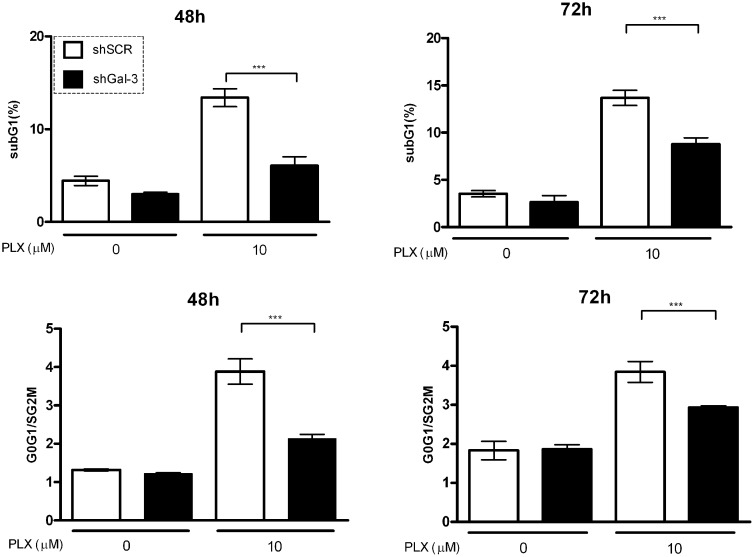
Galectin-3 silencing decreases both cell arrest in G0/G1 phases of the cell cycle and cell death induce by PLX in SK-MEL-37 cells Galectin-3 expression was downregulated using specific short-harpin RNA oligonucleotides (shGal-3). Cells transduced with a short-hairpin scrambled RNA sequence (shSCR) served as controls. Both cells were cultured either in the absence or presence of PLX for 48 and 72 h and then analyzed by flow cytometry. The proportion of dying cells (SubG1, upper panels) and the distribution of cells in different phases of cell cycle (ratio G0G1/SG2M, lower panels) was assessed using propidium iodide (PI) staining. All data are representative of four independent experiments in triplicate and are expressed as mean ± SD (*p* < 0.001, one way ANOVA, Bonferroni post-test).

Gal-3 is a multifunctional protein involved in melanoma progression and regulates several critical biological processes for melanoma development [27, 28]. To characterize the involvement of Gal-3 in the response of melanoma cells to the treatment with PLX-4032, a BRAF enzyme inhibitor used in the clinics to treat melanoma patients who have the BRAF^V600E^ mutation [29, 30], we first evaluated the distribution of cells in distinct cell cycle phases and cell death in Gal-3 silenced cells treated with PLX4032. As shown in Figure 1, in control cells (shSCR for scrambled short hairpin) PLX induced a G_0_/G_1_ phase arrest at 48 and 72 h after treatment. On the other hand, in shGal-3 cells PLX was not able to induce cell cycle arrest at 48 h. In relation to cell viability, PLX treatment induced more cell death in shSCR cells than in shGal-3 after 48 - 72 h, indicating that the expression of gal-3 is associated with relative resistance to cell death (Figure 1). Likewise, upon silencing of gal-3, SK-MEL-05 cells displayed the very same response to PLX, extending the finding observed in SK-MEL-37 cells (Supplementary Figure 4). However, the effects on SK-MEL-05 were attenuated in subsequent cell passaging.

### Galectin-3 regulates starvation-induced autophagic flux in melanocytes and melanoma cells

**Figure 2:**
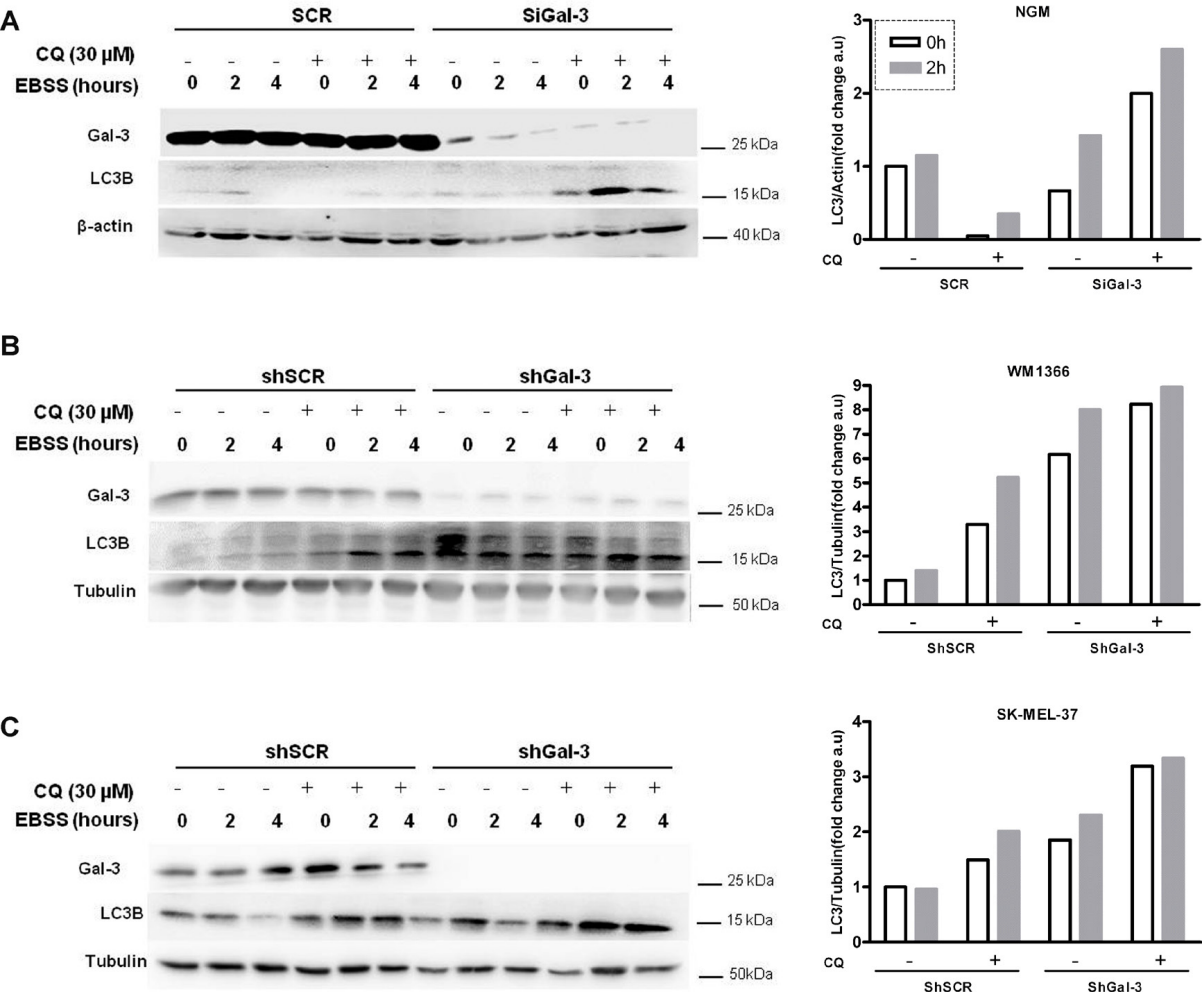
Galectin-3 acts as a negative regulator of starvation-induced autophagy in melanocytes and melanoma cells LC3 lipidation and galectin-3 expression were detected by western blotting in NGM melanocytes (**A**), WM1366 (**B**) and SK-MEL-37 (**C**) melanoma cells, modified with either scrambled (SCR or shSCR) or interference RNAs for galectin-3 (SiGal-3 or shGal-3). Cells under starvation (EBSS) were treated in the presence or absence of the lysosomal inhibitor chloroquine (CQ, 30 μM, and 1:30 h) at indicated times, as shown at each panel. Bar graphs represent the quantification of the Western blots for LC3B (LC3-II) normalized to either β-actin or tubulin of a representative assay of three independent experiments.

**Figure 3:**
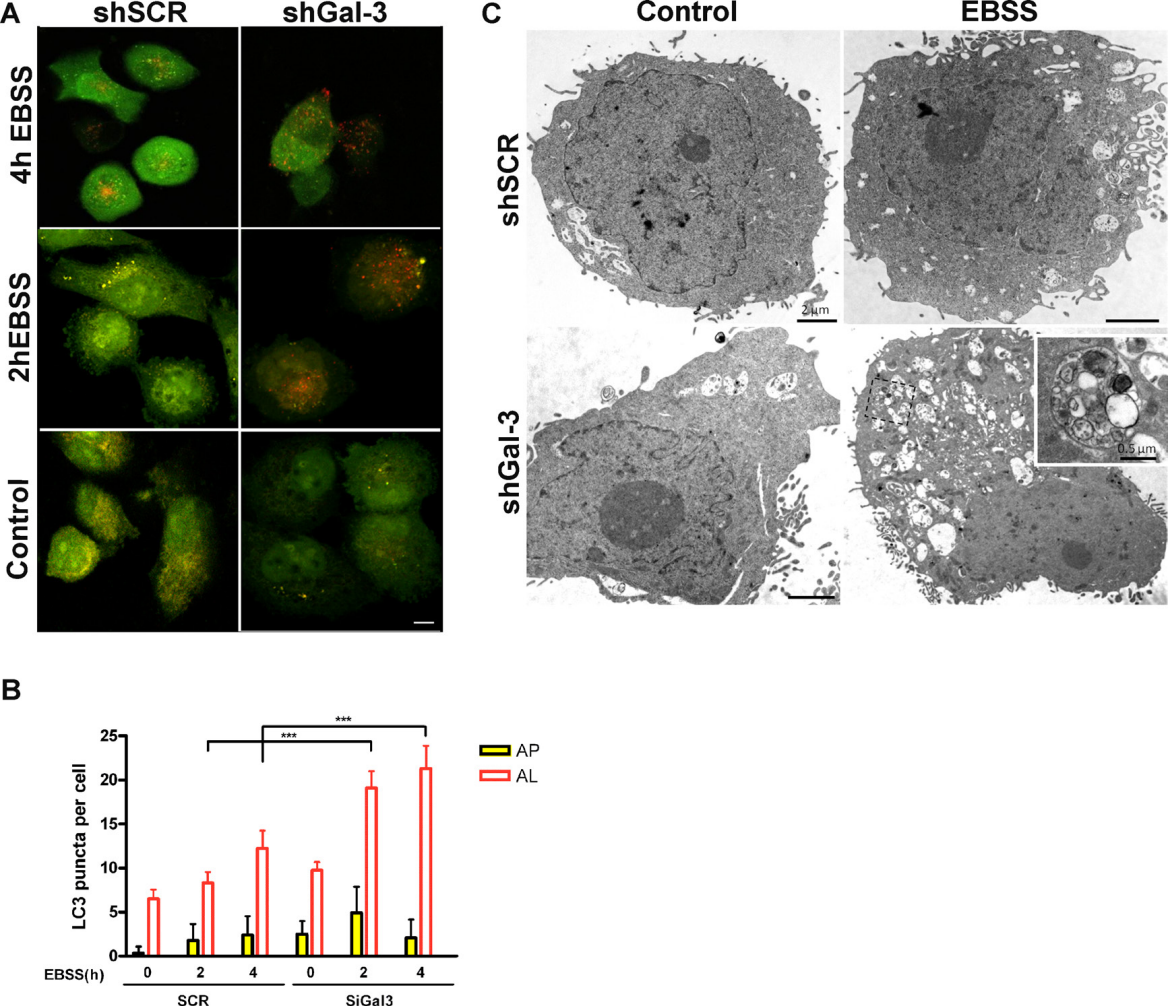
Galectin-3 inhibition increases autolysosome formation under starvation in WM1366 cells (**A**) Both shSCR and shGal-3 transduced WM1366 cells were transfected with a tandem fluorescent-tagged LC3 plasmid and further exposed to EBSS for 2 or 4 hours. Representative fluorescent image is shown (scale bars, 10 μm),(*n* = 2). (**B**) The autophagic flux was then analyzed in conditions indicated at each image by counting the number of GFP and mCherry puncta per cell. Autophagosomes (AP) are identified as positive puncta for both GFP and mCherry (yellow dots), autolysosomes (AL) are identified as mCherry-only positive puncta. Bars represent Mean ± SD, *p* < 0.001. (**C**) Ultrastructural images of melanoma cells (SK-MEL-37) treated with EBSS. Starvation (EBSS) induces vacuolar structures in melanoma cells after 2 h, which were more notable in shGal-3 cells. Several autophagic vacuoles with cytoplasmic cargo are presented (inset). Bars in the panoramic cell images represent 2 micra, while bar in the inset represents 0.5 micra.

Recently, Gal-3 has been postulated to cause endocytic trafficking and to be associated with damaged endomembrane, e.g. lysosomal membrane, recycling [31] [32]. Likewise, another member of the galectin family, galectin-8 was involved in autophagy in *Salmonella-*infected cells [33]. To better understand how Gal-3 might be associated with the autophagic flux mediated by starvation, assays to discriminate between the rate of autophagosome formation and lysosomal activity, following both LC3-II and p62 levels, were performed either in the presence or absence of lysosomal inhibitors, *e.g.* Chloroquine (30 μM). In these assays, NGM melanocytes and two melanoma cells (WM1366 and SK-MEL-37) were exposed to chloroquine in the last hour of starvation, harvested and analyzed. The levels of p62 were not significantly altered (data not shown), but LC3-II was accumulated overtime in Gal-3-silenced cells. For all cell lines studied, chloroquine further increased LC3-II levels in Gal-3-silenced cells, especially 2 h after starvation (Figure 2).The addition of chloroquine increased LC3-II levels in shSCR and shGal-3 cells independent of the treatment. Chloroquine combined with EBSS incremented even more the levels of LC3-II and such accumulation was more evident in the absence of Gal-3. Thereby, to monitor and compare the autophagic flux during starvation time between siSCR cells and Gal-3-silenced cells (siGal-3), WM1366 melanoma cells transfected with mCherry-eGFP-LC3 were used to assess the formation rate of autophagosomes (AF, defined by both cherry- and GFP-puncta, i.e. yellow puncta) and autolysosomes (AL, defined by cherry only-puncta, since GFP is quenched in low pH). Upon starvation, both cells exhibited increased number of autolysosomes after 4 h. However, Gal-3-silenced cells displayed higher density of autolysosome when compared to shSCR cells (Figure 3A–3B). Next we identified more precisely the presence of autophagosomes/autolysosomes by electron microscopy. Under starvation conditions, the ultrastructure of SK-MEL-37 cells revealed the presence of several autophagic vacuoles with double-membrane and electron-dense bodies (Figure 3C). Altogether, the data showed that Gal-3 inhibition increased the autophagic flux in melanoma cells under starvation.

### Inhibition of galectin-3 is related to basal LC3 expression in melanoma *in vivo*

**Figure 4:**
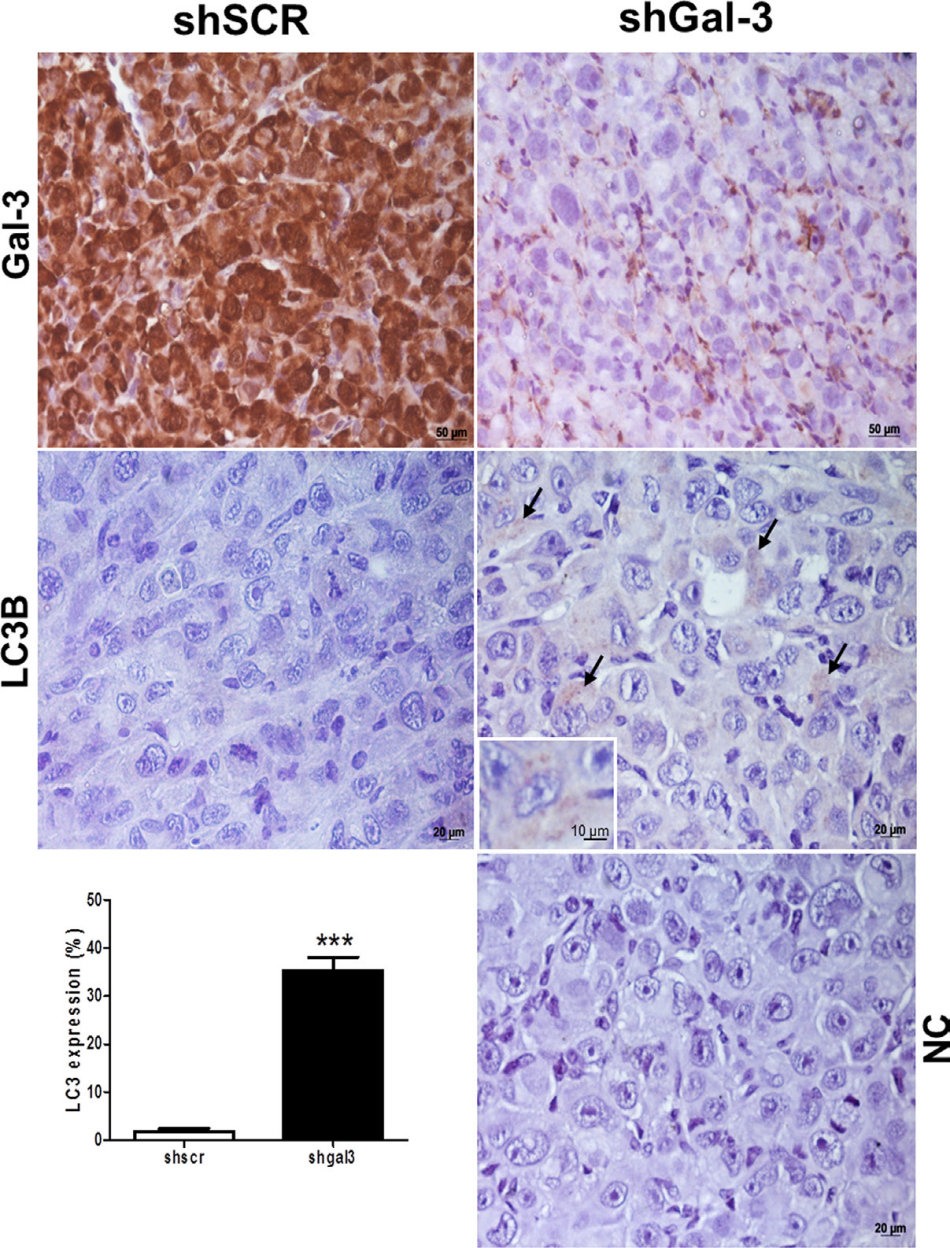
Galectin-3 and LC3B staining in melanoma tumors from mice inoculated with either shSCR or shGal-3 transduced SK-MEL-37 cells NC: negative control of shGal-3. Arrows indicate light brown-staining cells for the autophagy marker LC3B (LC3-II). In the bottom left, LC3B quantitative analysis by counting the proportion of positive cells. (*n* = 3). Error bars correspond to SD. (Student´s *T* test analysis, *p* < 0.001).

To further explore the basal LC3 levels in melanoma, SK-MEL-37 scramble or shGal-3 cells were injected subcutaneously in NOD/SCID mice and after 12 days, tumors were collected to detect Gal-3 and LC3-II by immunohistochemical staining. As shown in Figure 4, Gal-3 staining demonstrated that tumors derived from control shSCR-melanoma cells expressed Gal-3. However, in tumors derived from shGal-3-melanoma cells, Gal-3 was only observed in stromal cells (Figure 4, upper panel). Although basal LC3 staining was low in tumors, shGal-3 melanoma cells accumulated higher levels of LC3-II than Gal-3 expressing melanoma cells (Gal-3 ^high^; Figure 4, on the right). The LC3 quantification results were consistent with our previous *in vitro* data.

### Expression of Gal-3 determines the outcome of PLX-treatment in SK-MEL-37 human melanoma cells through autophagy

**Figure 5:**
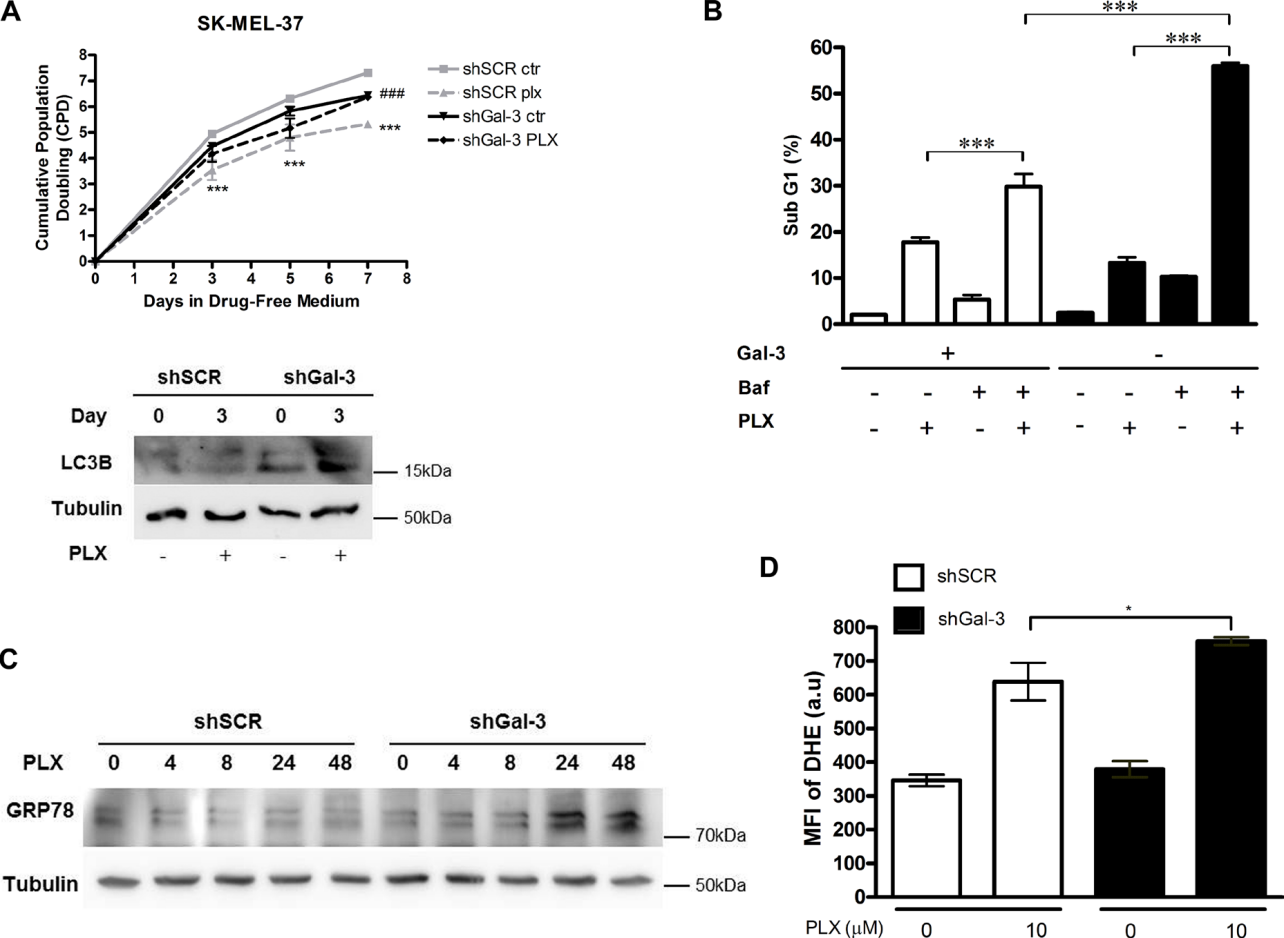
Expression of Gal-3 determines the outcome of PLX-treatment in SK-MEL-37 human melanoma cells through autophagy (**A**) Both shSCR and shGal-3 SK-MEL-37 cells were treated with PLX (10 μM) for 72 h and after 3,5 and 7 days the cumulative population doubling (CPD) was analyzed and the LC3 levels were detected in samples from days 0 and 3. While PLX was effective in reducing the CPD in shSCR cells (^***^shSCR PLX relative to shSCR ctr group, *p* < 0.001), no differences were observed in shGal-3 cells, which accumulated LC3B. PLX was effective in galectin-3 expressing cells, and not on Gal-3 silenced cells (###, shSCR PLX relative to shGal-3 PLX group, *p* < 0.001). (**B**) Autophagy inhibitor bafilomycin was added to the untreated or treated PLX cells and the analysis of cell death (SubG1) was performed by flow cytometry. Bafilomycin increased the sensitivity of both Gal-3 ^high^ and Gal-3^low/negative^ cells to PLX, reverting the relative resistance of Gal-3^low/negative^ as compared to Gal-3 ^high^ cells. (**C**) GRP78 was detected by western blotting in SK-MEL-37 cells treated with PLX after 4,8,24 and 48 h. Accumulation of GRP78 in later time points of PLX treatment of Gal-3^low/negative^ cells indicates activation of an endoplasmic reticulum stress response. (**D**) The mean fluorescence intensity (MFI) of the superoxide indicator dihydroethidium (DHE) was analyzed by flow cytometry after 48 h of PLX treatment.(a.u): arbitrary units. PLX led to increased superoxide accumulation on Gal-3^low/negative^ cells as compared to Gal-3 ^high^ cells. Data are represented as mean ± SD ^***^p < 0.001;^*^ *p* < 0.05. Figures show a representative assay from three independent experiments. Western blots were performed in two of the three experiments.

As Gal-3 ^high^ cells showed less LC3-II accumulation, as well as increased sensitivity to PLX-induced cell death, SK-MEL-37 cells were treated with PLX (10 μM) for 72 hours followed by growth in fresh medium for 3, 5 and 7 days. Interestingly, the cumulative population doubling (CPD) showed a significant difference between Gal-3 ^high^ cells, since PLX led to a decrease in proliferating cells, thus controlling cell growth. On the other hand, Gal-3-silenced cells continued growing actively after treatment (Figure 5A, upper). A western blot was performed to investigate LC3-II expression in the same samples (Figure 5A, bottom). shGal-3 cells accumulated more LC3-II after 3 days of PLX treatment, in agreement with previous results (Figure 2).

To explore whether the difference in cell death observed between shSCR and shGal-3 cells was related to autophagy we used two autophagy inhibitors: chloroquine (5 to 10 μM) and bafilomycin A1 (0.3 to 3 nM). Then, we evaluated the sub-G1 population after PLX treatment. We pre-treated the cells during 6 h with chloroquine or bafilomycin A1 to inhibit the last step of autophagy and avoid LC3-II degradation. Under these conditions, the cells were treated with PLX4032 for 48 h then cell death analyses were performed. As shown in Figure 5B our results evidenced that pre-treatment with either bafilomycin or chloroquine (not shown) in combination with PLX significantly potentiated cell death, as compared to the PLX effect alone. Furthermore, the addition of Baf to PLX reduced the number of viable cells in shGal-3 group (Supplementary Figure 5). These observations suggest that the inhibitors are able to reverse the phenomena, previously demonstrated in Figure 1, via the inhibition of lysosome acidification and autophagy blockade. Together, these data showed that Gal-3 inhibition regulates autophagy and in turn this process interferes in the PLX response to cell death in shGal-3 cells.

Since melanoma is associated with ER stress and also with oxidative stress, we further tested the levels of GRP78, one of the most important ER-resident chaperones, and measured the superoxide generation in PLX-treated cells. The results revealed that PLX was capable to increasing GRP78 levels in shGal-3 cells after 48 h (Figure 5C). In the same context, the detection of superoxide by dihydroethidium (DHE) showed that PLX induces oxidative stress and such effect is greater in the shGal-3 group (Figure 5D). Because ROS is increased in the shGal-3 group, we further used an antioxidant, apocynin, to verify if LC3B accumulation observed in Gal-3 silenced cells is dependent on ROS levels. We showed that upon inhibition of NADPH oxidase activity, LC3B levels decreased after 48 h in both groups and interestingly, apocynin also reduced Gal-3 expression in shSCR cells, indicating a tight correlation between oxidative stress state and Gal-3 expression (Supplementary Figure 3). Altogether, our results showed that (i) the cell stress machinery was activated by PLX treatment of melanoma cells; (ii) autophagy protected melanoma cells from PLX-induced cell death; (iii) the autophagic flux rate was increased in the absence of Gal-3 conferring an advantage to Gal-3-silenced cells to survive to PLX treatment.

## DISCUSSION

In the last years, autophagy has been widely studied as a critical metabolic process involved in cancer development, cancer cell survival and therefore in its resistance to treatment. Autophagy is often upregulated in melanoma [34] and Gal-3, a pleiotropic protein related to several cancer pathways, is down-regulated during melanoma progression [35]. Recently, a few studies showed that Gal-3 plays a role in autophagy, however, the mechanisms remain unclear. Understanding the role of Gal-3 in melanoma and as it relates with autophagy will generate new insights to autophagy pathways in cancer.

In the present work, we found that Gal-3 in melanocytes and melanoma cells decreased autophagy activity and it also regulated the cell fate after the treatment with the mutated BRAF inhibitor, vemurafenib (PLX-4032 or PLX). Moreover, autophagy blockage sensitizes melanoma cells to PLX treatment. Although PLX is used to treat BRAF^V600E^ melanoma, patients regularly develop resistance to the drug, due to different mechanisms such as alternative activation of ERK signaling and autophagy induction [34, 36]. Firstly, we tested the effect of PLX on shSCR (Gal-3 ^high^) and shGal-3 (Gal-3^low/negative^) SK-MEL-37 cells and demonstrated that Gal-3 expressing cells were more sensitive to PLX than Gal-3 ^low/negative^ cells. We next evaluated the autophagic flux assessing LC3-II and p62 levels in both Gal-3 ^high^ and Gal-3^low/negative^ cells. Interestingly, Gal-3^low/negative^ melanocytes and melanoma cells accumulated LC3-II after 2 h of starvation and chloroquine addition. Several studies showed that advanced malignant melanomas have increased levels of LC3-II as compared to their counterparts in early phases of progression [37]. Here, we show, for the first time, that loss of Gal-3 favors accumulation of LC3-II. Under these conditions, we were not able to detect changes in the levels of p62, a scaffold protein required for the aggregation of ubiquitinylated proteins [38, 39]. Failure in detecting a decrease in p62 levels in this study could be due to the fact that p62 (sequestosome-1) is regulated at transcriptional level, by different pathways, and its basal expression can be quickly restored after starvation [40, 41].

Increasing evidence associates Gal-3 with the autophagic process. Gal-3 is involved with the recruitment of the autophagic machinery and it is also important to recruit membranes to the autophagosome formation [42–44]. In this context, Gal-3 is important to the recognition of damaged endomembranes. In addition, Chauhan and collaborators demonstrated that TRIM16 interacts with Gal-3 in a ULK-1 dependent manner acting as a platform to organize autophagy factors and activate stages of selective autophagy in response to lysosomal and phagosomal damage [45]. The discrepancies between our results, related to increased autophagy in Gal-3^low/negative^ cells is at least in part explained by the autophagy induction protocol we exploited herein, based on starvation and not directly dependent on endomembrane damage, as those mentioned above. Furthermore, it is well known that Gal-3 may play several roles depending on its subcellular location, where it regulates many fundamental biological processes involved in signaling, protein-protein interactions, metabolism, survival and cell death [1, 46, 47].

As a homeostatic process, autophagy is usually a transient response to different acute stressors. However, cancer cells maintain higher levels of autophagic markers chronically, as a survival adaptation strategy [48–50]. Additionally, other works using oncogene overexpression or viral infection showed a delay in autophagy induction [51, 52]. Based in these data and our findings, the differences in the CPD profile of Gal-3 ^high^ and Gal-3^low/negative^ cells upon PLX treatment were related with up-regulation of autophagy in Gal-3^low/negative^ cells. As autophagy may protect tumor cells through tumor cell adaptation and therefore resistance to stress, it could be necessary for the growth maintenance of Gal-3^low/negative^ cells despite the treatment with PLX, as observed in Figure 5A and Supplementary Figure 1.

Autophagy may be regulated by several stress stimuli and their effectors, such as hypoxia, nutrient starvation, chemical drugs, viral infection, AMPK, ER stress, Erk1/2, ATP/AMP radio or intracellular reactive oxygen species (ROS) [53]. ROS have been described among the main intracellular signal transducers involved in autophagy induction. ROS, such as hydrogen peroxide (H_2_O_2_) [54] and the superoxide anion (O_2_^−^) [55] are upstream modulators of autophagy acting as oxidative stressors and inducers of cellular damage. ROS initiate early steps of autophagy and autophagy also reduces ROS levels through removal of proteins and damaged organelles [56]. Defects in autophagy promote accumulation of damaged structures leading to increase oxidative stress, thereby assembling an environment, which contributes to cancer development [57, 58]. Moreover, oxidative stress production in tumor cells in the microenvironment can induce autophagy in stroma cells stimulating metabolism and proliferation of cancer cells [59].

ER stress, as a cellular response to ROS, is an important regulator of autophagy as reviewed elsewhere [60]. ER stress is caused by the accumulation of misfolded or damaged proteins in the ER lumen. Such stress activates the unfolded protein response (UPR), which interferes with ER-resident chaperone levels, like GRP78, and ROS generation [61–63]. Accordingly, we observed higher levels of superoxide production in Gal-3^low/negative^ cells after 48 h of PLX treatment. Upon accumulation of superoxide, Gal-3^low/negative^ cells also accumulated GRP78 after 48 h of PLX treatment (Figure 5C and 5D). Taken together, these data indicate that Gal-3 protects cells from oxidative stress, as initially suggested by its homeostatic role in mitochondria [64]. In the absence of Gal-3, cells are in a pro-oxidative state and their survival would rely on autophagy. While it is clear that autophagy contributes to the cell fate, there is a dual response, survival or death, dependent on the stimulus and the signaling involved [65, 66]. In our setting of SK-MEL-37 cells, we observed a differential cell death response between Gal-3^low/negative^ and Gal-3 ^high^ cells. Importantly, blockage of autophagy by late autophagy inhibitors sensitized both groups of cells after PLX treatment indicating that the pathway involved in the differential response to PLX is autophagy, acting as a survival mechanism in this context.

Considering the close integration among all these pathways and MAPK signaling as well as several reports that exhibit the regulation of autophagy in BRAF mutant melanoma, we hypothesized that extracellular signal-regulated kinase (ERK) may contribute to the regulation of autophagy in our model. Additionally, Gal-3 has been associated several times with different molecules that participate in the Ras–Raf–MAPK cascade [67–69]. Interestingly, a recent study identifies a new role for Gal-3 in which Gal-3 interaction with ανβ3 integrin drives cancer cell addiction to oncogenic KRAS. In this study, blocking Gal-3 extracellular function with a neutralizing pectin that competes for Gal-3 binding reduced nutrient uptake and increased ROS levels [70] in cancer cells. Here, we showed that the expression levels of pERK were increased in Gal-3^low/negative^ cells after 48 h of PLX treatment (Supplementary Figure 2). Therefore, despite the inhibition of BRAF^V600E^, Gal-3^low/negative^ cells rapidly rewired the RAS-RAF-MAPK pathway.

Maintenance of the activation of the ERK pathway under specific inhibitors, such as PLX, is critical for the emergence of resistant cells. In this regard, it has been shown that activation of autophagy (ATG) proteins led to the organization of molecular platforms that maintain the activation status of ERK [71] Collectively, the increased levels of ROS, ER-stress and activation of autophagy-related pathways in Gal-3^low/negative^ cells defined the surviving phenotype in melanoma cells. On the other hand, as a negative regulator of autophagy, Gal-3 expression in tumor cells sensitizes them to the therapeutic effect of PLX. Analysis of Gal-3 expression in cohorts of patients under vemurafenib use will be useful to test whether Gal-3 could be used as a proxy for efficacy of ERK pathway inhibitors in melanoma.

## MATERIALS AND METHODS

### Cell culture

Human SK-MEL-37 melanoma cells (mutated *BRAF*) were cultured in MEM (Invitrogen). SK-MEL-05 human (mutated *BRAF*) and WM1366 melanoma cells (wild-type *BRAF*) were cultured in DMEM (Invitrogen) supplemented with 10% fetal bovine serum (FBS, Gibco), respectively. NGM cell line, derived from melanocytes of blue nevus, was obtained from Cell Bank of Rio de Janeiro (Brazil), cultured in DMEM supplemented with FBS 20% and HGMS (Invitrogen). SK-MEL-37 cells were kindly provided by Ludwig Cancer Research Institute. WM1366 and SK-MEL-05 cells were donated by Marisol Soengas (Centro Nacional de Investigaciones Oncológicas, Madrid, Spain). All cultured cells were maintained in a humidified incubator with 5% CO_2_ at 37°C.

### Transient transfection and gal-3 specific siRNA

For SK-MEL-37 and WM1366 Gal-3 shRNA knockdown, cells were transduced with a lentivirus containing a *LGALS3* shRNA sequence (OpenBiosystems, pLKO-shGAL-3 Cat. TRC0000029305) and then subjected to cell selection by puromycin (1 μg/mL) (Gibco). The shRNA scramble sequence was used as a transduction control. Gal-3 knockdown was evaluated by western blot in different passages. The plasmidial vector pLPCX (Clontech) tandem-tagged LC3 construct mCherry-eGFP-LC3 was used as previously described in Grasso *et al.* (2016) [72]. For gal-3 siRNA knockdown assays, WM1366 cells were transfected using a mix containing Lipofectamine ^®^ RNAiMAX (Invitrogen), opti-MEM medium (Gibco, Invitrogen) and 20 μM of gal-3 siRNA (5′UUU CCU GAU UAG UGC UCC ACC CGC CGC-3′; 5′-GGG GGG UGG AGC ACU AAU CAG GAA A-3′) or 20 μM of scramble siRNA (IDT, Coralville, IA). NGM cells were electroporated (1200 V, 10 ms, 3 p) using Neon transfection system (Invitrogen) and 20 μM of gal-3 siRNA or 20 μM of scramble siRNA (IDT, Coralville, IA).

### Cell treatments

To evaluate LC3 levels, cells were treated with chloroquine 30 μM (CQ, Sigma-Aldrich) for 90 minutes before the end the experiment. For the cell death experiments, cells were pretreated with 10 μM of CQ for 6 h and then 10 μM of PLX-4032 (Selleck, USA) in DMSO in combination with 5 μM of CQ. The same conditions were used in experiments with bafilomycin (BAF, Tocris Bioscience, UK) using BAF 3 nM and reduced to 0.5 nM after 6 h. Starvation was induced by EBBS Earle's Balanced Salt Solution (Calcium Chloride 1.8 mM, Potassium Chloride 5.3 mM, Magnesium Sulfate 0.8 mM, Sodium Chloride 117 mM, Sodium Bicarbonate 26 mM and Sodium Phosphate Monobasic 1 mM). To reduce ROS levels we treated the cells with apocynin 100 μM during 2, 24 and 48 hours. The cumulative population doubling was performed as described in Filippe-Chiela *et al.* [50].

### DNA content analysis by propidium iodide staining (PI)

All the cells were cultured in triplicates in 24-well plates (2.10^4^ cells) and exposed to CQ, BAF or PLX as described above. After 48 or 72 h, cells were analyzed for their DNA content by PI staining for the evaluation of sub-G1 population and the distribution of cells in the distinct phases of cell cycle. Cells were harvested by trypsinization, fixed in ethanol 70% and analyzed by flow cytometry (Attune, Invitrogen). Next, the cells were washed with PBS, centrifuged for 4 min at 2000 rpm and incubated in 20 μg/mL of propidium iodide, 200 μg/mL of RNAse A and 0,1% v:v of Triton X-100 in PBS for 30 minutes protected from light. The cellular DNA content was analyzed using the Attune software.

### Western blot

Cells were cultured in 6-well plates (2.5.10^5^ cells/well), where they were exposed to the described treatment conditions. After treatment, the cells were lysed in a buffer containing 50 mM Tris, pH 7.5, 150 mM NaCl, 10 mM MgCl2, 0.5 mM DTT, 1 mM EDTA, 10% glycerol, 2% SDS, 1% Triton X-100 and protease inhibitors (aprotinin 2 μg/mL and PMSF 1 mM) for 30 min on ice. After centrifugation (13.000 *rpm* for 15 min at 4°C), the protein supernatants were collected, quantified by the Bradford method (Bio-Rad, Richmond, CA) and 30 μg total proteins were separated by SDS-PAGE. Next, the proteins were transferred onto PVDF membranes, blocked with 5% non-fat dry milk or TBS/BSA 5% for 1 h and incubated overnight with primary antibodies: LC3B (1:1000, Cell Signalling Technologies, Beverly, MA, USA, 2775), M3/38 rat antiGal-3 (1:100), GRP78 (1:1000, N-20, Santa Cruz Biotechnology, sc-1050), ERK and pERK from Sigma (Rehovot, Israel). For loading controls, antibodies to β-actin (1:4000, Sigma-Aldrich, Saint Louis, MO, USA, AC-74), GAPDH (1:4000, Ambion Thermo Fischer Scientific, Waltham, MA, USA, AM4300) and Tubulin (1:1000, Calbiochem, USA, DM1A) were used. The samples were visualized with the chemiluminescent substrate ECL (GE Healthcare). When necessary, we stripped the membranes using a buffer containing 25 mM glycine-HCl, pH 2, and 1% SDS. Spot intensity measurement was performed using ImageJ^®^ software.

### Confocal microscopy assay

WM1366 cells expressing mCherry-eGFP-LC3 were grown on coverslips, starved with EBSS and fixed with 4% paraformaldehyde for 15 min in PBS. Coverslips were mounted and the autophagic flux was measured under a confocal microscope (Zeiss 710, Germany).

### Electron microscopy

SK-MEL-37 cells were starved with EBSS and later fixed in a 2% paraformaldehyde, 2% glutaraldehyde solution (1:1) in sodium cacodylate buffer 0.1 M (pH 7.4) for 24 h at 4°C. The cells were subsequently post-fixed in 1% osmium tetroxide, dehydrated in a graded ethanol series and embedded in Araldite resin (Electron Microscopy Science). Ultrathin sections (~90 nm) were stained with uranyl acetate and lead citrate and examined using a ZEISS Leo 906 electron microscope (Carl Zeiss).

### Animals and immunohistochemistry

All procedures were in accordance with the guidelines of the Brazilian Council on Animal Care (COBEA) and approved by the Ethical Committee for Animal Research of School of Medicine, University of São Paulo. Eight-week-old female NOS/SCID wild-type (WT) mice were divided into two groups: shSCR (*n* = 6) and shGal-3 (*n* = 6). These animals were kept in sterile and specific pathogen free environments supplied with water and certified rodent diet *ad libitum*. Mice received a subcutaneous inoculation of 5 × 10^5 SK-MEL-37^ cells in right flank and after 12 days euthanasia was done and tumors were collected for immunohistochemistry. Immunohistochemistry was performed on paraffin sections from SK-MEL-37 tumors. Sections were stained with rabbit LC3 (1:100, Cell Signalling Technologies, Beverly, MA, USA, 2775), rat Gal-3 (1:500, Santa Cruz Biotechnology, Texas, USA) diluted in 1% BSA overnight. After washing, the specimens were incubated with a secondary biotinylated antibody (LAB-SA Detection kit, Invitrogen, Paisley, UK). Positive staining was detected using a peroxidase-conjugated streptavidin complex, and color was developed using DAB substrate (Invitrogen). The sections were counterstained with hematoxylin and analyzed in Axioskop 2-Mot Plus Zeiss microscope (Carl Zeiss). To quantify LC3 stain positive cells were counted in different fields and the percentage of stain cells were calculated. Negative controls for immunohistochemistry consisted of specimens incubated only with the secondary biotinylated antibody and further developed.

### Detection of reactive oxygen species (ROS) by Dihydroethidium (DHE)

DHE is useful for the detection of superoxide. Cells were plated in 24-well plates (2 × 10^4^) and treated with PLX for 48 h. After harvesting, at least 10^5^ viable cells from each experimental condition were incubated with 4 μM DHE in HBSS for 30 minutes at 37°C and then immediately analyzed using a flow cytometer.

### Statistical analysis

All statistical analyses were performed with the GraphPad Prism 5.0 software. Differences were considered statistically significant if the probability value was less than 0.05 and all data are presented as mean ± SD.^*^ *P* ≤ 0.05, ^**^ *P* ≤ 0.01,^***^ *P* ≤ 0.001.

## SUPPLEMENTARY MATERIALS FIGURES AND TABLES



## Abbreviations

PLX4032: Vemurafenib
Gal-3: Galectin-3
ROS: Reactive Oxygen Species
UPR: Unfolded Protein Response
MAPK: Mitogen-Activated Protein Kinase
ATG: autophagy-related
CPD: cumulative population doubling

## References

[1] Cardoso AC, Andrade LN, Bustos SO, Chammas R (2016). Galectin-3 Determines Tumor Cell Adaptive Strategies in Stressed Tumor Microenvironments. Front Oncol.

[2] Thijssen VL, Heusschen R, Caers J, Griffioen AW (2015). Galectin expression in cancer diagnosis and prognosis: A systematic review. Biochim Biophys Acta.

[3] Ruvolo PP (2016). Galectin 3 as a guardian of the tumor microenvironment. Biochim Biophys Acta.

[4] Lepur A, Carlsson MC, Novak R, Dumić J, Nilsson UJ, Leffler H (2012). Galectin-3 endocytosis by carbohydrate independent and dependent pathways in different macrophage like cell types. Biochim Biophys Acta.

[5] Wang L, Guo XL (2016). Molecular regulation of galectin-3 expression and therapeutic implication in cancer progression. Biomed Pharmacother.

[6] Anaka M, Hudson C, Lo PH, Do H, Caballero OL, Davis ID, Dobrovic A, Cebon J, Behren A (2013). Intratumoral genetic heterogeneity in metastatic melanoma is accompanied by variation in malignant behaviors. BMC Med Genomics.

[7] Elder DE (2016). Melanoma progression. Pathology.

[8] Winder M, Virós A (2017). Mechanisms of Drug Resistance in Melanoma. Handb Exp Pharmacol.

[9] Brown ER, Doig T, Anderson N, Brenn T, Doherty V, Xu Y, Bartlett JM, Smyth JF, Melton DW (2012). Association of galectin-3 expression with melanoma progression and prognosis. Eur J Cancer.

[10] Li ZW, Wang Y, Xue WC, Si L, Cui CL, Cao DF, Zhou LX, Guo J, Lu AP (2013). [Expression and prognostic significance of galectin-1 and galectin-3 in benign nevi and melanomas]. Zhonghua Bing Li Xue Za Zhi.

[11] Demirsoy S, Martin S, Maes H, Agostinis P (2016). Adapt, Recycle, and Move on: Proteostasis and Trafficking Mechanisms in Melanoma. Front Oncol.

[12] Amaral T, Sinnberg T, Meier F, Krepler C, Levesque M, Niessner H, Garbe C (2017). MAPK pathway in melanoma part II-secondary and adaptive resistance mechanisms to BRAF inhibition. Eur J Cancer.

[13] Kon M, Kiffin R, Koga H, Chapochnick J, Macian F, Varticovski L, Cuervo AM (2011). Chaperone-mediated autophagy is required for tumor growth. Sci Transl Med.

[14] Thorburn A, Thamm DH, Gustafson DL (2014). Autophagy and cancer therapy. Mol Pharmacol.

[15] White E (2015). The role for autophagy in cancer. J Clin Invest.

[16] Parzych KR, Klionsky DJ (2014). An overview of autophagy: morphology, mechanism, and regulation. Antioxid Redox Signal.

[17] Abada A, Elazar Z (2014). Getting ready for building: signaling and autophagosome biogenesis. EMBO Rep.

[18] Liang XH, Jackson S, Seaman M, Brown K, Kempkes B, Hibshoosh H, Levine B (1999). Induction of autophagy and inhibition of tumorigenesis by beclin 1. Nature.

[19] Towers CG, Thorburn A (2016). Therapeutic Targeting of Autophagy. EBioMedicine.

[20] Lippai M, Szatmári Z (2017). Autophagy-from molecular mechanisms to clinical relevance. Cell Biol Toxicol.

[21] Mowers EE, Sharifi MN, Macleod KF (2017). Autophagy in cancer metastasis. Oncogene.

[22] Hara Y, Nakamura M (2012). Overexpression of autophagy-related beclin-1 in advanced malignant melanoma and its low expression in melanoma-in-situ. Eur J Dermatol.

[23] Maes H, Martin S, Verfaillie T, Agostinis P (2014). Dynamic interplay between autophagic flux and Akt during melanoma progression *in vitro*. Exp Dermatol.

[24] Lazova R, Klump V, Pawelek J (2010). Autophagy in cutaneous malignant melanoma. J Cutan Pathol.

[25] Corazzari M, Fimia GM, Lovat P, Piacentini M (2013). Why is autophagy important for melanoma? Molecular mechanisms and therapeutic implications. Semin Cancer Biol.

[26] Ndoye A, Weeraratna AT (2016). Autophagy- An emerging target for melanoma therapy. F1000Res.

[27] Mourad-Zeidan AA, Melnikova VO, Wang H, Raz A, Bar-Eli M (2008). Expression profiling of Galectin-3-depleted melanoma cells reveals its major role in melanoma cell plasticity and vasculogenic mimicry. Am J Pathol.

[28] Wang YG, Kim SJ, Baek JH, Lee HW, Jeong SY, Chun KH (2012). Galectin-3 increases the motility of mouse melanoma cells by regulating matrix metalloproteinase-1 expression. Exp Mol Med.

[29] Bollag G, Tsai J, Zhang J, Zhang C, Ibrahim P, Nolop K, Hirth P (2012). Vemurafenib: the first drug approved for BRAF-mutant cancer. Nat Rev Drug Discov.

[30] Shelledy L, Roman D (2015). Vemurafenib: First-in-Class BRAF-Mutated Inhibitor for the Treatment of Unresectable or Metastatic Melanoma. J Adv Pract Oncol.

[31] Paz I, Sachse M, Dupont N, Mounier J, Cederfur C, Enninga J, Leffler H, Poirier F, Prevost MC, Lafont F, Sansonetti P (2010). Galectin-3, a marker for vacuole lysis by invasive pathogens. Cell Microbiol.

[32] Lakshminarayan R, Wunder C, Becken U, Howes MT, Benzing C, Arumugam S, Sales S, Ariotti N, Chambon V, Lamaze C, Loew D, Shevchenko A, Gaus K (2014). Galectin-3 drives glycosphingolipid-dependent biogenesis of clathrin-independent carriers. Nat Cell Biol.

[33] Thurston TL, Wandel MP, von Muhlinen N, Foeglein A, Randow F (2012). Galectin 8 targets damaged vesicles for autophagy to defend cells against bacterial invasion. Nature.

[34] Ma XH, Piao SF, Dey S, McAfee Q, Karakousis G, Villanueva J, Hart LS, Levi S, Hu J, Zhang G, Lazova R, Klump V, Pawelek JM (2014). Targeting ER stress-induced autophagy overcomes BRAF inhibitor resistance in melanoma. J Clin Invest.

[35] Brauer M, Amann M, Burnett RT, Cohen A, Dentener F, Ezzati M, Henderson SB, Krzyzanowski M, Martin RV, Van Dingenen R, van Donkelaar A, Thurston GD (2012). Exposure assessment for estimation of the global burden of disease attributable to outdoor air pollution. Environ Sci Technol.

[36] Villanueva J, Vultur A, Lee JT, Somasundaram R, Fukunaga-Kalabis M, Cipolla AK, Wubbenhorst B, Xu X, Gimotty PA, Kee D, Santiago-Walker AE, Letrero R, D’Andrea K (2010). Acquired resistance to BRAF inhibitors mediated by a RAF kinase switch in melanoma can be overcome by cotargeting MEK, IGF-1R/PI3K. Cancer Cell.

[37] Tang DY, Ellis RA, Lovat PE (2016). Prognostic Impact of Autophagy Biomarkers for Cutaneous Melanoma. Front Oncol.

[38] Pankiv S, Clausen TH, Lamark T, Brech A, Bruun JA, Outzen H, Øvervatn A, Bjørkøy G, Johansen T (2007). p62/SQSTM1 binds directly to Atg8/LC3 to facilitate degradation of ubiquitinated protein aggregates by autophagy. J Biol Chem.

[39] Rusten TE, Stenmark H (2010). p62, an autophagy hero or culprit?. Nat Cell Biol.

[40] Moscat J, Diaz-Meco MT (2009). p62 at the crossroads of autophagy, apoptosis, and cancer. Cell.

[41] Sahani MH, Itakura E, Mizushima N (2014). Expression of the autophagy substrate SQSTM1/p62 is restored during prolonged starvation depending on transcriptional upregulation and autophagy-derived amino acids. Autophagy.

[42] Chen X, Khambu B, Zhang H, Gao W, Li M, Yoshimori T, Yin XM (2014). Autophagy induced by calcium phosphate precipitates targets damaged endosomes. J Biol Chem.

[43] Fujita N, Morita E, Itoh T, Tanaka A, Nakaoka M, Osada Y, Umemoto T, Saitoh T, Nakatogawa H, Kobayashi S, Haraguchi T, Guan JL, Iwai K (2013). Recruitment of the autophagic machinery to endosomes during infection is mediated by ubiquitin. J Cell Biol.

[44] Maejima I, Takahashi A, Omori H, Kimura T, Takabatake Y, Saitoh T, Yamamoto A, Hamasaki M, Noda T, Isaka Y, Yoshimori T (2013). Autophagy sequesters damaged lysosomes to control lysosomal biogenesis and kidney injury. EMBO J.

[45] Chauhan S, Kumar S, Jain A, Ponpuak M, Mudd MH, Kimura T, Choi SW, Peters R, Mandell M, Bruun JA, Johansen T, Deretic V (2016). TRIMs and Galectins Globally Cooperate and TRIM16 and Galectin-3 Co-direct Autophagy in Endomembrane Damage Homeostasis. Dev Cell.

[46] Song L, Tang JW, Owusu L, Sun MZ, Wu J, Zhang J (2014). Galectin-3 in cancer. Clin Chim Acta.

[47] Nabi IR, Shankar J, Dennis JW (2015). The galectin lattice at a glance. J Cell Sci.

[48] Mizushima N (2007). Autophagy: process and function. Genes Dev.

[49] Wojtkowiak JW, Rothberg JM, Kumar V, Schramm KJ, Haller E, Proemsey JB, Lloyd MC, Sloane BF, Gillies RJ (2012). Chronic autophagy is a cellular adaptation to tumor acidic pH microenvironments. Cancer Res.

[50] Filippi-Chiela EC, Bueno e Silva MM, Thomé MP, Lenz G (2015). Single-cell analysis challenges the connection between autophagy and senescence induced by DNA damage. Autophagy.

[51] Wu SY, Lan SH, Cheng DE, Chen WK, Shen CH, Lee YR, Zuchini R, Liu HS (2011). Ras-related tumorigenesis is suppressed by BNIP3-mediated autophagy through inhibition of cell proliferation. Neoplasia.

[52] Ito H, Aoki H, Kühnel F, Kondo Y, Kubicka S, Wirth T, Iwado E, Iwamaru A, Fujiwara K, Hess KR, Lang FF, Sawaya R, Kondo S (2006). Autophagic cell death of malignant glioma cells induced by a conditionally replicating adenovirus. J Natl Cancer Inst.

[53] Li L, Tan J, Miao Y, Lei P, Zhang Q (2015). ROS, Autophagy: Interactions and Molecular Regulatory Mechanisms. Cell Mol Neurobiol.

[54] Scherz-Shouval R, Shvets E, Elazar Z (2007). Oxidation as a post-translational modification that regulates autophagy. Autophagy.

[55] Chen Y, Azad MB, Gibson SB (2009). Superoxide is the major reactive oxygen species regulating autophagy. Cell Death Differ.

[56] Filomeni G, De Zio D, Cecconi F (2015). Oxidative stress and autophagy: the clash between damage and metabolic needs. Cell Death Differ.

[57] Kongara S, Karantza V (2012). The interplay between autophagy and ROS in tumorigenesis. Front Oncol.

[58] Poillet-Perez L, Despouy G, Delage-Mourroux R, Boyer-Guittaut M (2015). Interplay between ROS and autophagy in cancer cells, from tumor initiation to cancer therapy. Redox Biol.

[59] Zhou W, Xu G, Wang Y, Xu Z, Liu X, Xu X, Ren G, Tian K (2017). Oxidative stress induced autophagy in cancer associated fibroblast enhances proliferation and metabolism of colorectal cancer cells. Cell Cycle.

[60] Zeeshan HM, Lee GH, Kim HR, Chae HJ (2016). Endoplasmic Reticulum Stress and Associated ROS. Int J Mol Sci.

[61] Li J, Ni M, Lee B, Barron E, Hinton DR, Lee AS (2008). The unfolded protein response regulator GRP78/BiP is required for endoplasmic reticulum integrity and stress-induced autophagy in mammalian cells. Cell Death Differ.

[62] Bhandary B, Marahatta A, Kim HR, Chae HJ (2012). An involvement of oxidative stress in endoplasmic reticulum stress and its associated diseases. Int J Mol Sci.

[63] Senft D, Ronai ZA (2015). UPR, autophagy, and mitochondria crosstalk underlies the ER stress response. Trends Biochem Sci.

[64] Matarrese P, Tinari N, Semeraro ML, Natoli C, Iacobelli S, Malorni W (2000). Galectin-3 overexpression protects from cell damage and death by influencing mitochondrial homeostasis. FEBS Lett.

[65] Yang ZJ, Chee CE, Huang S, Sinicrope FA (2011). The role of autophagy in cancer: therapeutic implications. Mol Cancer Ther.

[66] Das G, Shravage BV, Baehrecke EH (2012). Regulation and function of autophagy during cell survival and cell death. Cold Spring Harb Perspect Biol.

[67] Elad-Sfadia G, Haklai R, Balan E, Kloog Y (2004). Galectin-3 augments K-Ras activation and triggers a Ras signal that attenuates ERK but not phosphoinositide 3-kinase activity. J Biol Chem.

[68] Saegusa J, Hsu DK, Liu W, Kuwabara I, Kuwabara Y, Yu L, Liu FT (2008). Galectin-3 protects keratinocytes from UVB-induced apoptosis by enhancing AKT activation and suppressing ERK activation. J Invest Dermatol.

[69] Gao X, Balan V, Tai G, Raz A (2014). Galectin-3 induces cell migration via a calcium-sensitive MAPK/ERK1/2 pathway. Oncotarget.

[70] Seguin L, Camargo MF, Wettersten HI, Kato S, Desgrosellier JS, von Schalscha T, Elliott KC, Cosset E, Lesperance J, Weis SM, Cheresh DA (2017). Galectin-3, a druggable vulnerability for KRAS-addicted cancers. Cancer Discov.

[71] Martinez-Lopez N, Athonvarangkul D, Mishall P, Sahu S, Singh R (2013). Autophagy proteins regulate ERK phosphorylation. Nat Commun.

[72] Grasso S, Pereira GJ, Palmeira-Dos-Santos C, Calgarotto AK, Martínez-Lacaci I, Ferragut JA, Smaili SS, Bincoletto C (2016). Autophagy regulates Selumetinib (AZD6244) induced-apoptosis in colorectal cancer cells. Eur J Med Chem.

