# Supplementary material for: Galectin-3 sensitized melanoma cell lines to vemurafenib (PLX4032) induced cell death through prevention of autophagy
Source: Oncotarget. 2018 Feb 16;9(18):14567–79. doi: 10.18632/oncotarget.24516 (PMC5865690; doi:10.18632/oncotarget.24516)
Supplement: Supplementary file 1 [file oncotarget-09-14567-s001.pdf]

Galectin-3 sensitized melanoma cell lines to vemurafenib (PLX4032) induced cell death through prevention of autophagy

SUPPLEMENTARY MATERIALS

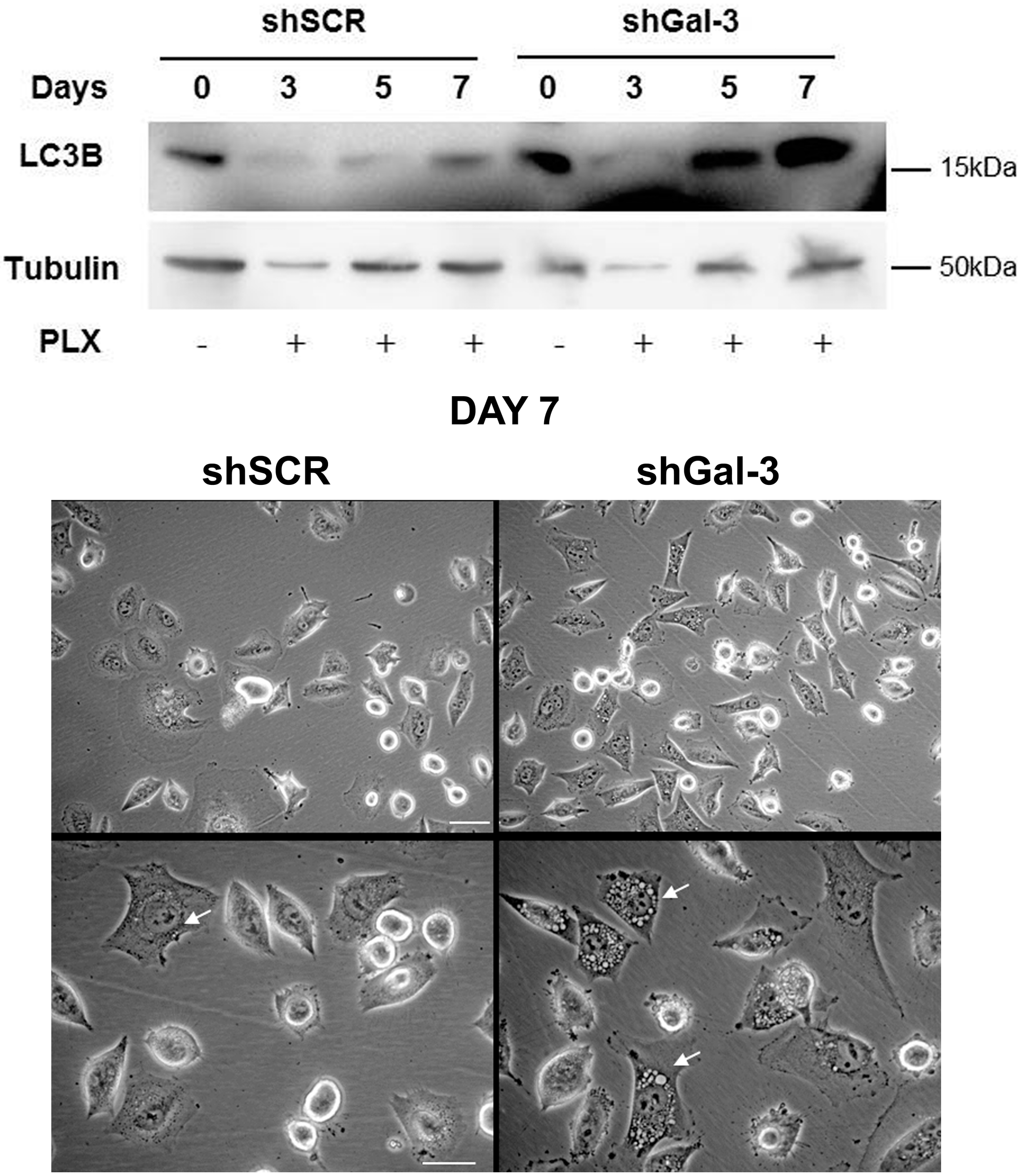

Supplementary Figure 1: PLX treatment impaired cell growth after 7 days in shSCR SK-MEL-37 cells. Upper: Detection of LC3B in samples from the CPD assay (described in Figure 5). Cell growth was followed by 3, 5 and 7 days and 90 minutes before cell harvesting in each time point, 30 μM chloroquine was added. Accumulation of LC3B (LC3-II) was evident in Gal-3$^{low/negative}$. Lower panel: phase-contrast micrographs of PLX SK-MEL-37 cells. Micrographs showed the morphology of cells after 7 days of treatment. Note the accumulation of intracellular vacuoles, which were more prominent in Gal-3$^{low/negative}$ after 7 days of PLX treatment. Scale bars, 10 μm.

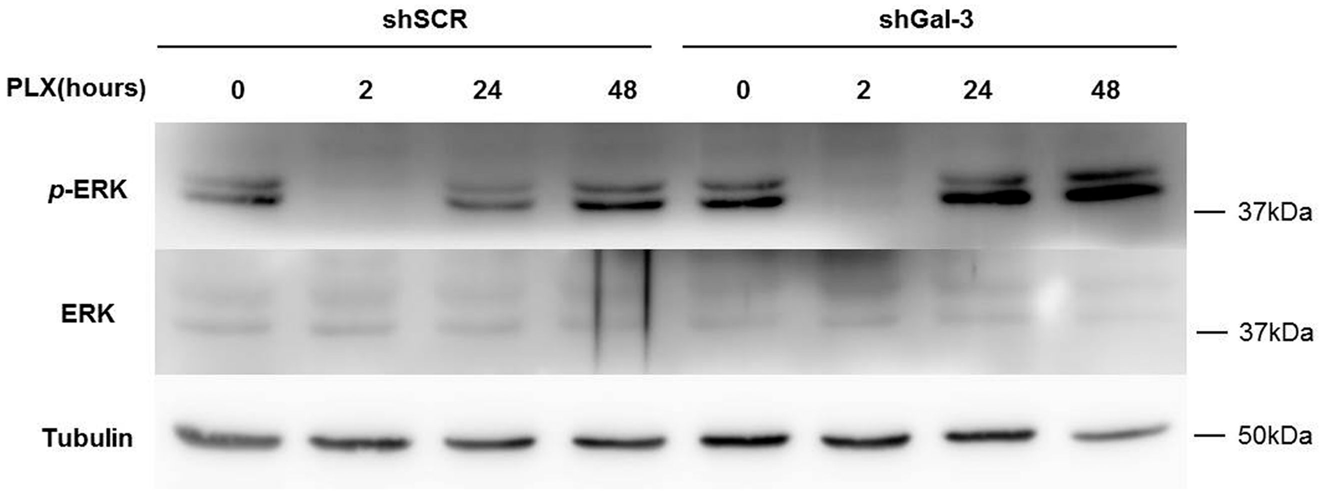

Supplementary Figure 2: Rewiring of ERK pathway activation in the presence of PLX. SK-MEL-37 cells treated with PLX (10 µM), which actively inhibited ERK activation in 2 hours. However, after 24 and 48 h reactivation of ERK was more evident in Gal-3$^{low/negative}$ cells, as compared to Gal-3^{high} cells.

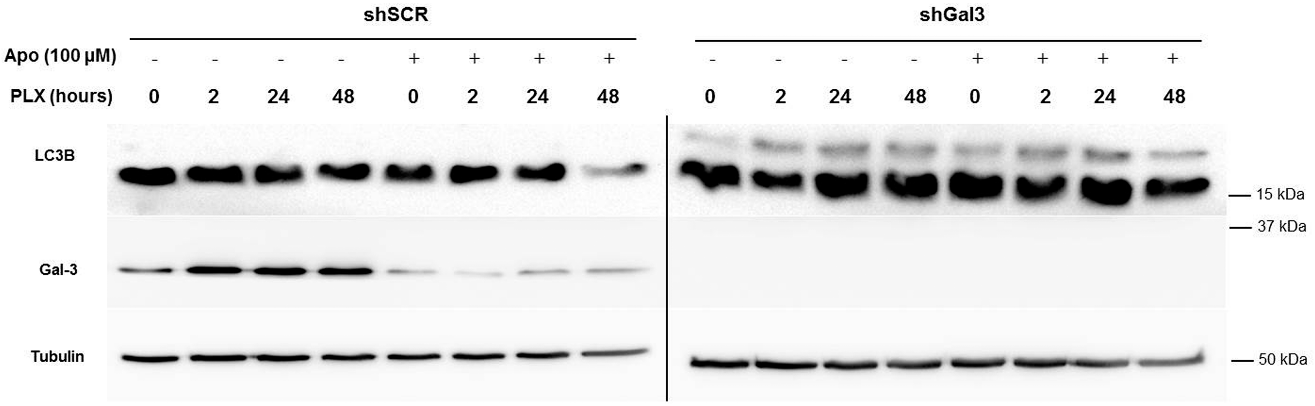

Supplementary Figure 3: The NADPH-oxidase inhibitor apocynin reduces the levels of both LC3B and galectin-3 in SK-MEL-37. shSCR (left) and shGal-3 cells (right) were treated with PLX (10 µM), and apocynin (100 µM) for 2-48 hours, as indicated. Cell extracts were then separated by SDS-PAGE, transferred onto PVDF membranes and used for detection of LC3B and Gal-3 in SK-MEL-37. Upon inhibition of NADPH-oxidase, reduced oxidative stress was followed by a decrease in the stimulus for autophagy, as observed by decreased conversion of LC3 into LC3B-II. Note that upon oxidative stress alleviation by NADPH-oxidase, there was also less accumulation of gal-3 in gal-3 expressing cells.

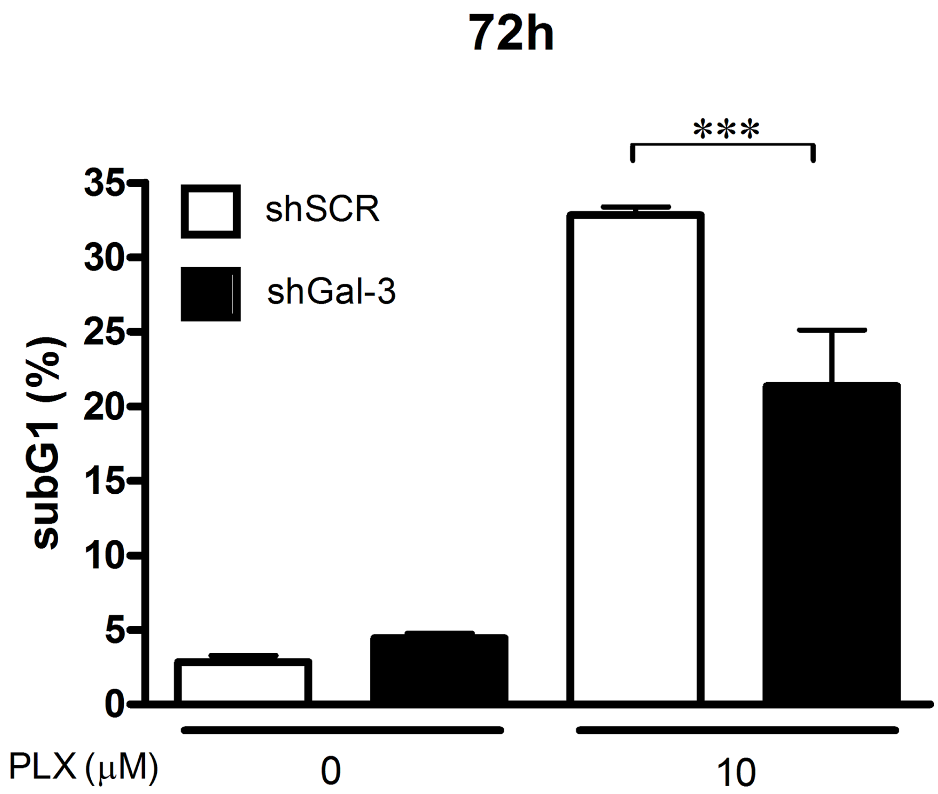

Supplementary Figure 4: Galectin-3 silencing decreases cell death induced by PLX in SK-MEL-05 cells. The proportion of dying cells (SubG1) was assessed using propidium iodide (PI) staining after 72h of PLX 4032 treatment, as described in previous figures.

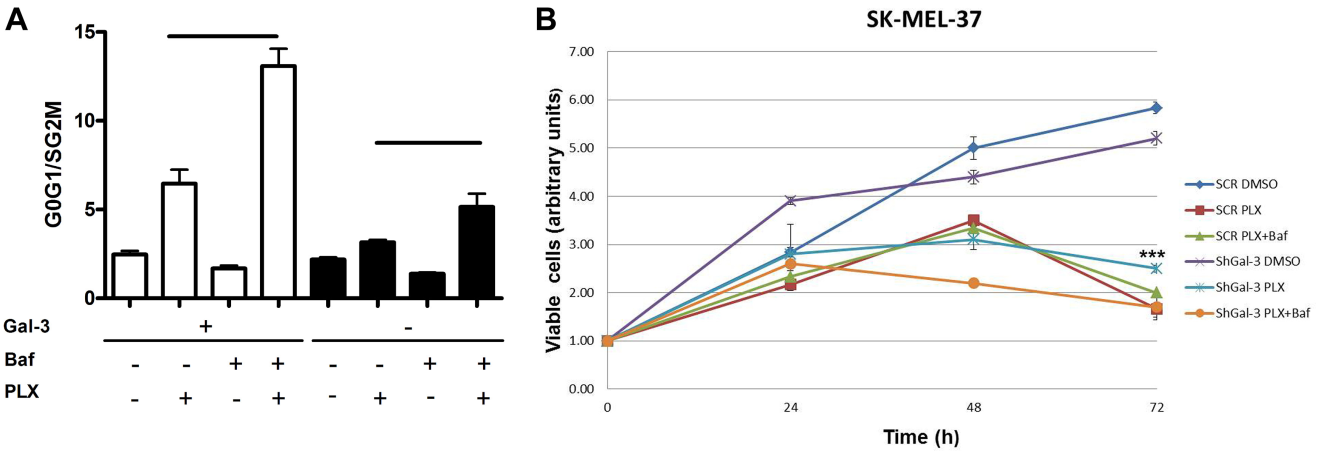

Supplementary Figure 5: The autophagy inhibitor bafilomycin increased cell arrest in G0/G1 phases and reduced SK-MEL-37 cell growth. Bafilomycin was added to the untreated or treated PLX cells and the analyses of both cell cycle and cell growth were performed (**A** and **B**, respectively). Bafilomycin induced cell arrest in G0/G1 phases in both scr-SK-MEL-37 and shGal-3-SK-MEL-37 cells and delayed cell growth after the combination of PLX in shGal-3 cells, but not in scr-cells. Black lines and ***$p < 0.001$, two-way ANOVA, followed by Bonferroni post-test.
